# Supplementary material for: Cell fate specification modes shape transcriptome evolution in the highly conserved spiral cleavage
Source: EMBO Rep. 2025 Sep 4;26(20):5088–114. doi: 10.1038/s44319-025-00569-4 (PMC12550047; doi:10.1038/s44319-025-00569-4)
Supplement: Supplementary file 1 — Appendix [file 44319_2025_569_MOESM1_ESM.pdf]

Appendix

Cell-fate specification modes guide transcriptome evolution in the highly conserved spiral cleavage

Yan Liang, Jingcheng Wei, Yue Kang, Allan M. Carrillo-Baltodano, José M. Martín-Durán

Index

	Page
• Appendix Figure S1 – Quality check of the transcriptomic time course during *O. fusiformis* spiral cleavage.	3
• Appendix Figure S2 – Quality check of the transcriptomic time course during *C. teleta* spiral cleavage.	4
• Appendix Figure S3 – Gene Ontology enrichment for upregulated genes in *O. fusiformis*.	5
• Appendix Figure S4 – Gene Ontology enrichment for downregulated genes in *O. fusiformis*.	6
• Appendix Figure S5 – Gene Ontology enrichment for upregulated genes in *C. teleta*.	7
• Appendix Figure S6 – Gene Ontology enrichment for downregulated genes in *C. teleta*.	8
• Appendix Figure S7 – Codon usage during spiral cleavage.	9
• Appendix Figure S8 – Functional annotation of clusters of temporally co-regulated genes in *O. fusiformis*.	10
• Appendix Figure S9 – Functional annotation of clusters of temporally co-regulated genes in *C. teleta*.	11

- Appendix Figure S10 – The proportion of transcription factors during spiral cleavage. 13
- Appendix Figure S11 – Gene Ontology enrichment in maternal genes in *O. fusiformis*. 14
- Appendix Figure S12 – Gene Ontology enrichment in maternal genes in *C. teleta*. 15
- Appendix Figure S13 – Gene Ontology enrichment of genes exhibiting heterochronic shifts between different spiralian taxa. 16
- Appendix Table S1 – Number of up- and downregulated genes during early embryogenesis in *O. fusiformis* and *C. teleta*. 17
- Appendix Table S2 – Sequencing and mapping statistics for *O. fusiformis*. 18
- Appendix Table S3 – Sequencing and mapping statistics for *C. teleta*. 19
- Appendix Table S4 – Primers used to clone the validated transcription factors in *O. fusiformis* and *C. teleta*. 20

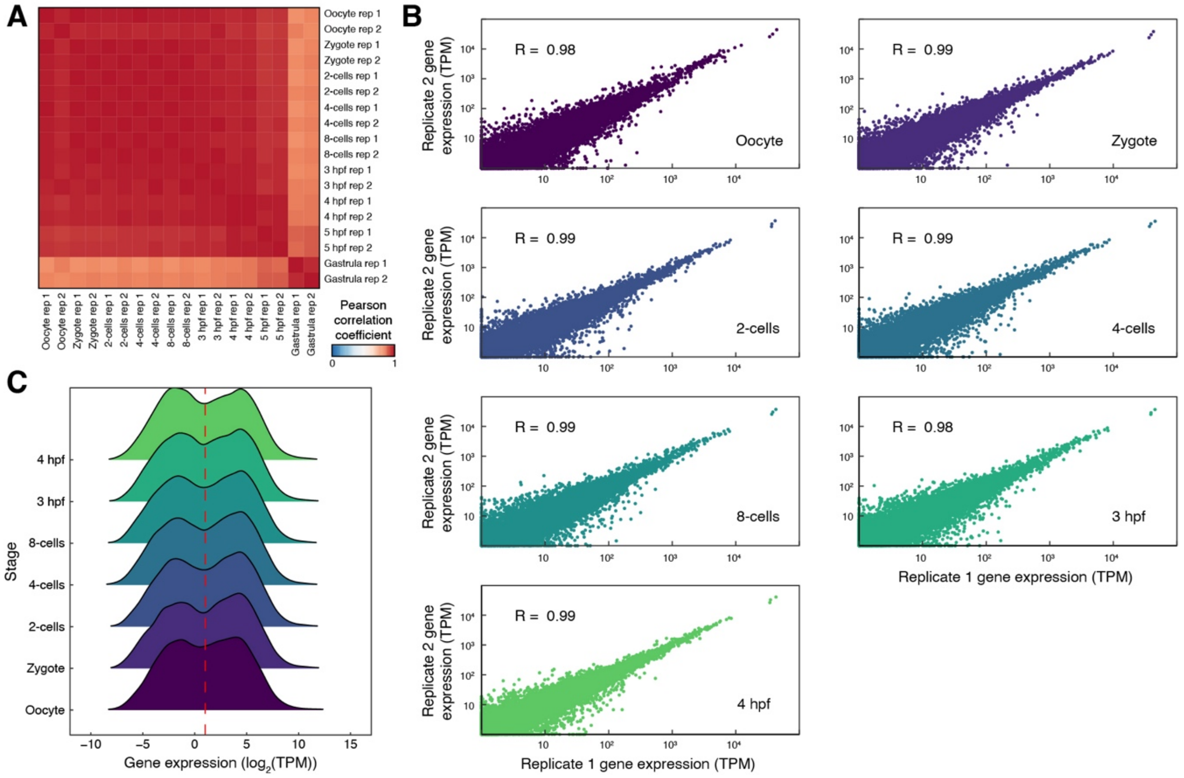

Appendix Figure S1 – Quality check of the transcriptomic time course during *O. fusiformis* spiral cleavage. (**A**) Pearson correlation coefficient matrix of the samples. Cleavage stages are highly similar to each other and distinct from the gastrula. (**B**) Dot plots of replicate-to-replicate correlation. Replicates for all stages are highly correlated. (**C**) Distribution plots of transcript per million (TPM) values during early spiral cleavage. TPM = 2 (vertical red dotted line) appears to demarcate the transition between expressed and non-expressed.

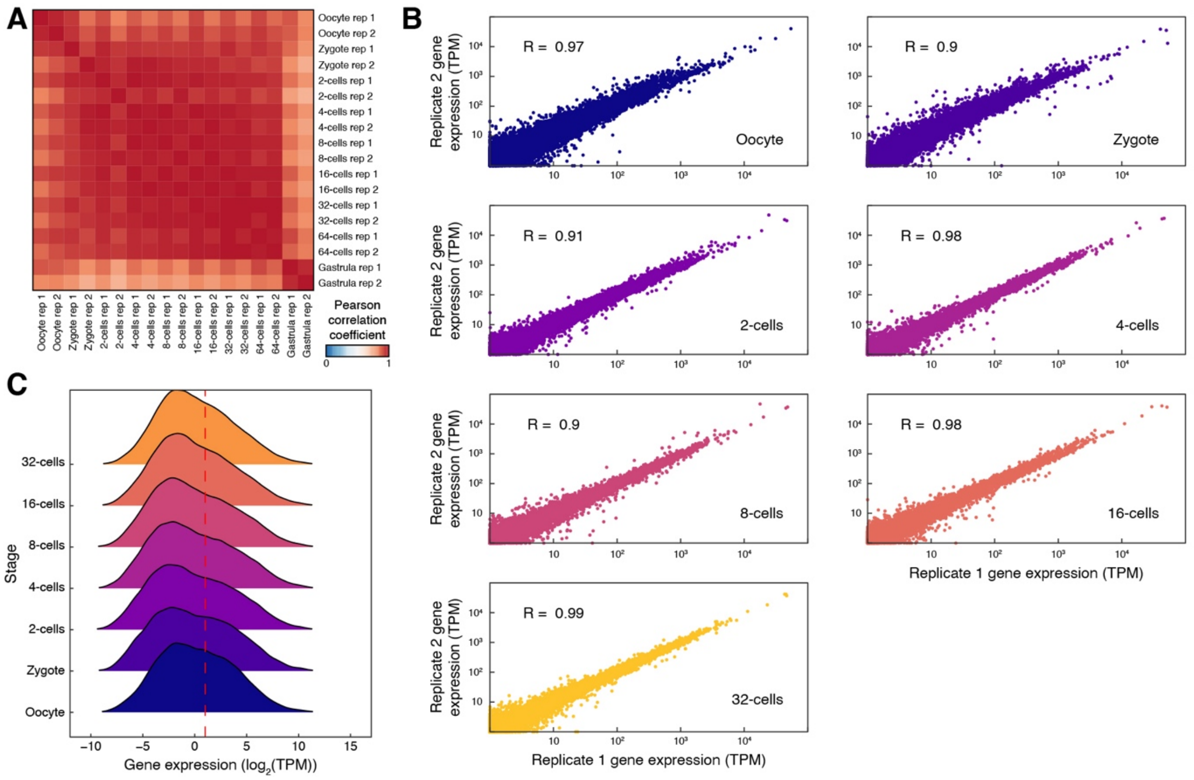

Appendix Figure S2 – Quality check of the transcriptomic time course during *C. teleta* spiral cleavage. (**A**) Pearson correlation coefficient matrix of the samples. Cleavage stages are highly similar to each other and distinct from the gastrula. (**B**) Dot plots of replicate-to-replicate correlation. Replicates for all stages are highly correlated. (**C**) Distribution plots of transcript per million (TPM) values during early spiral cleavage. TPM = 2 (vertical red dotted line) appears to demarcate the transition between expressed and non-expressed.

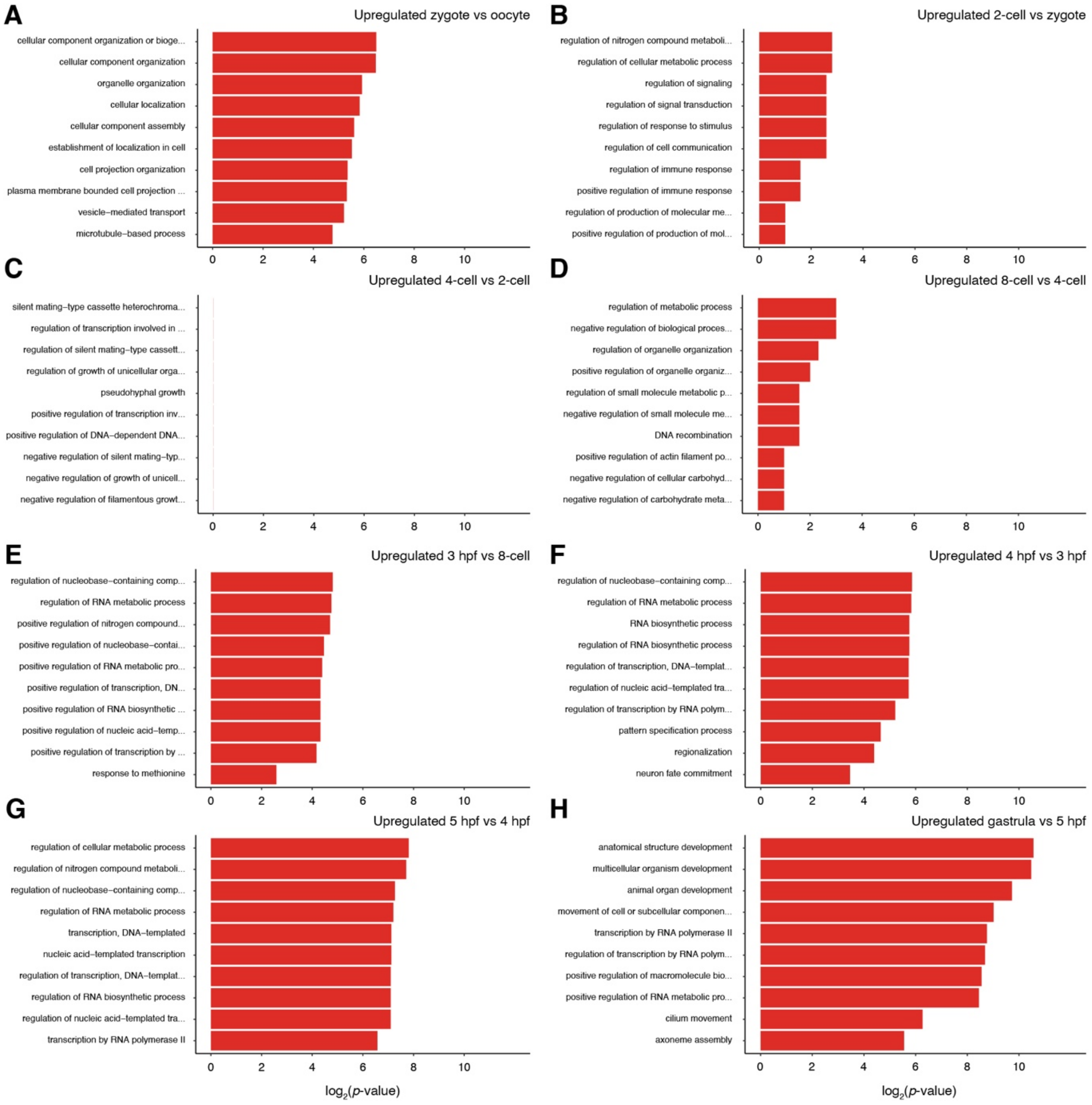

Appendix Figure S3 – Gene Ontology enrichment for upregulated genes in *O. fusiformis*. (**A–H**) Bar plots indicating the top ten Gene Ontology (GO) categories amongst upregulated genes in each consecutive pairwise comparison during spiral cleavage in *O. fusiformis*.

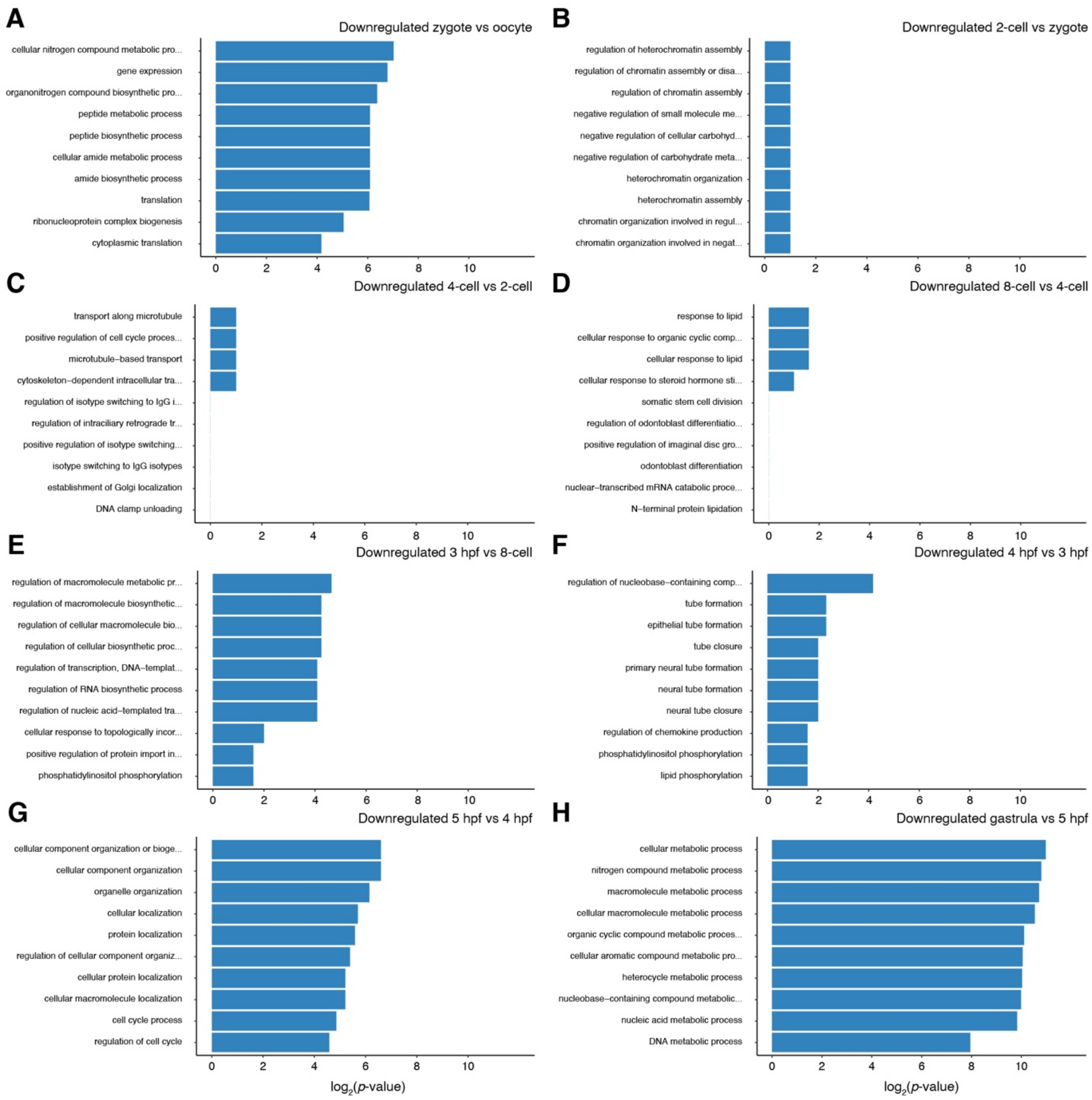

Appendix Figure S4 – Gene Ontology enrichment for downregulated genes in *O. fusiformis*. (**A–H**) Bar plots indicating the top ten Gene Ontology (GO) categories amongst downregulated genes in each consecutive pairwise comparison during spiral cleavage in *O. fusiformis*.

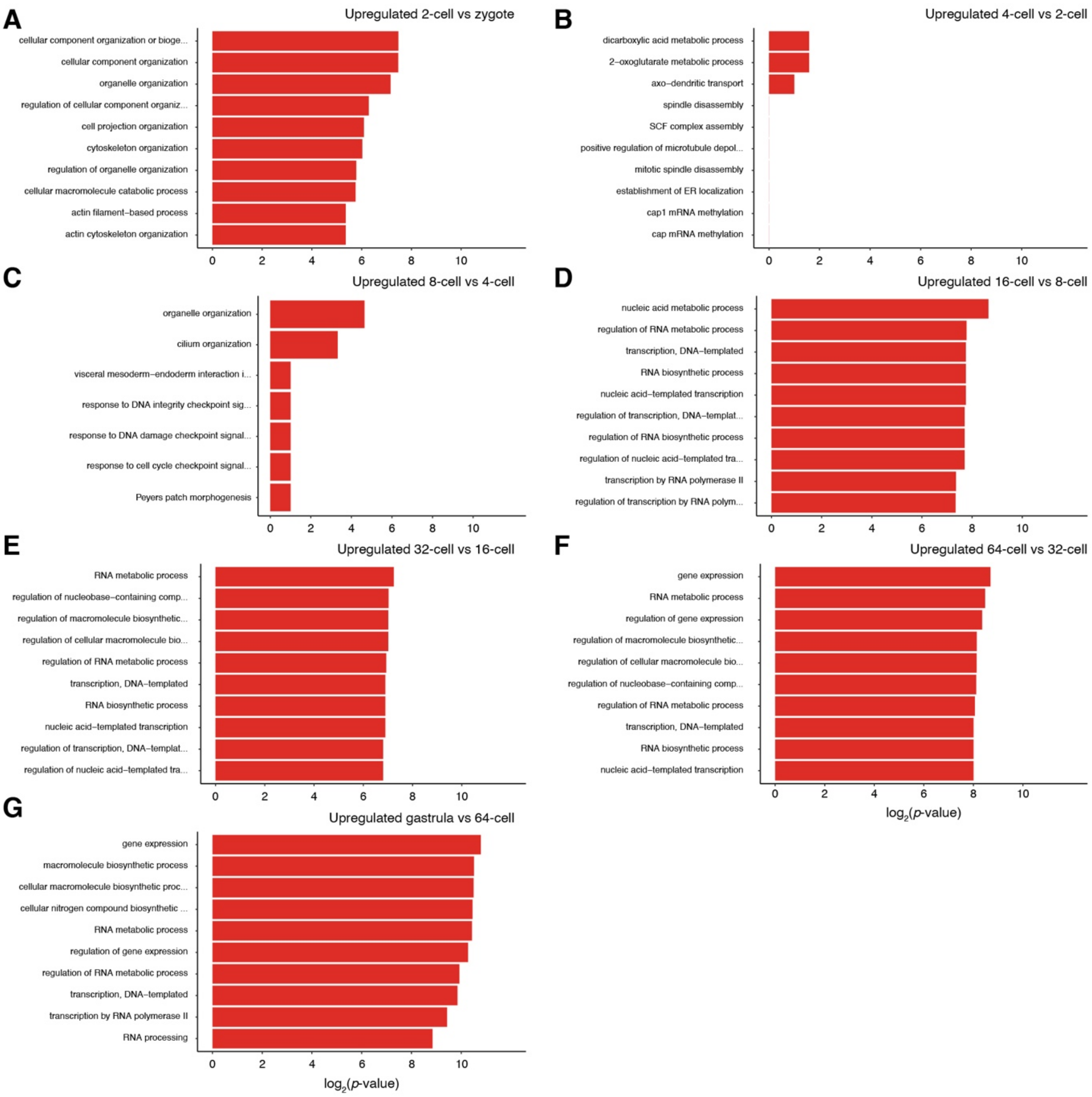

Appendix Figure S5 – Gene Ontology enrichment for upregulated genes in *C. teleta*. (**A–G**) Bar plots indicating the top ten Gene Ontology (GO) categories amongst upregulated genes in each consecutive pairwise comparison during spiral cleavage in *C. teleta*.

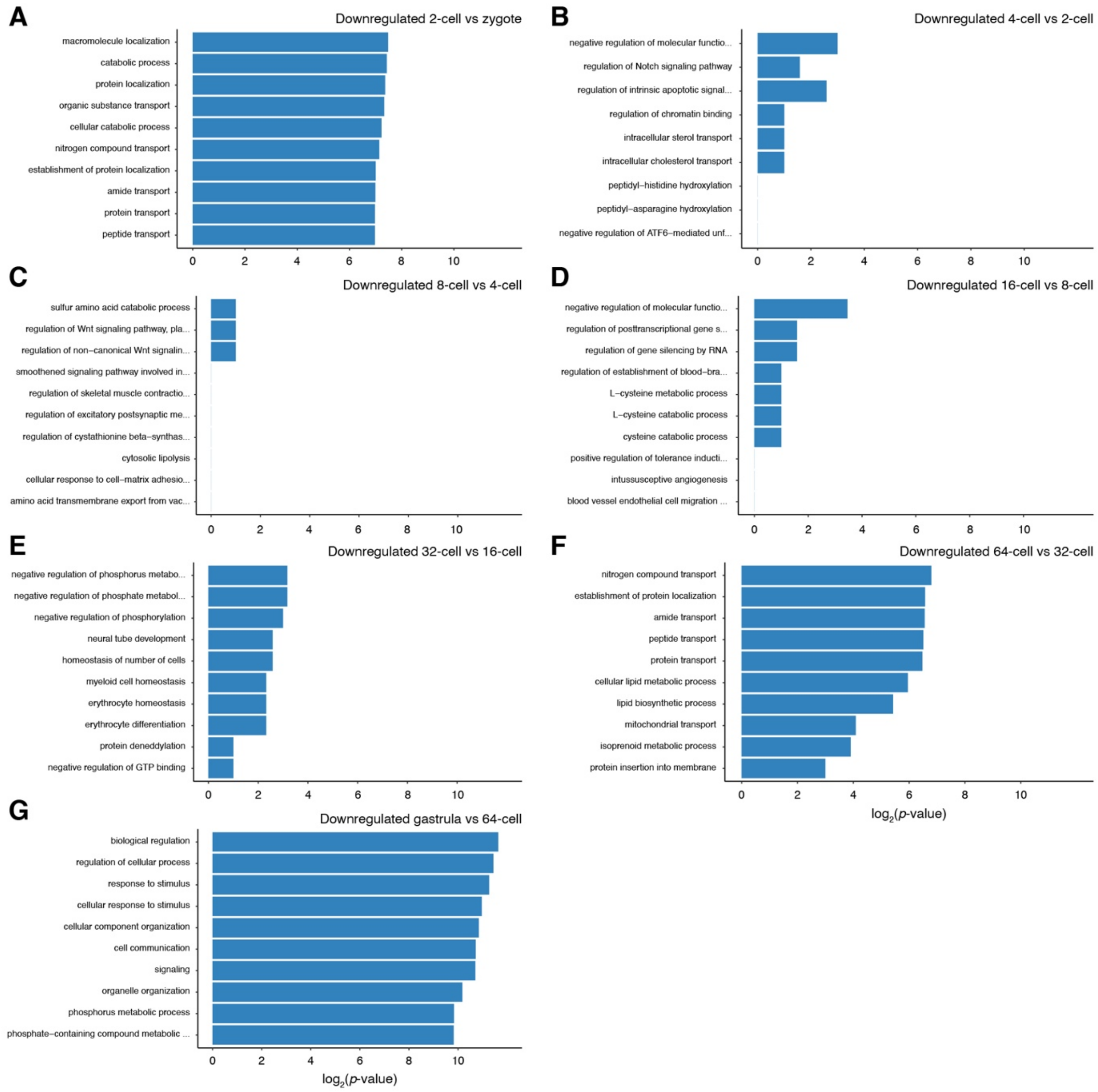

Appendix Figure S6 – Gene Ontology enrichment for downregulated genes in *C. teleta*. (**A–G**) Bar plots indicating the top ten Gene Ontology (GO) categories amongst downregulated genes in each consecutive pairwise comparison during spiral cleavage in *C. teleta*.

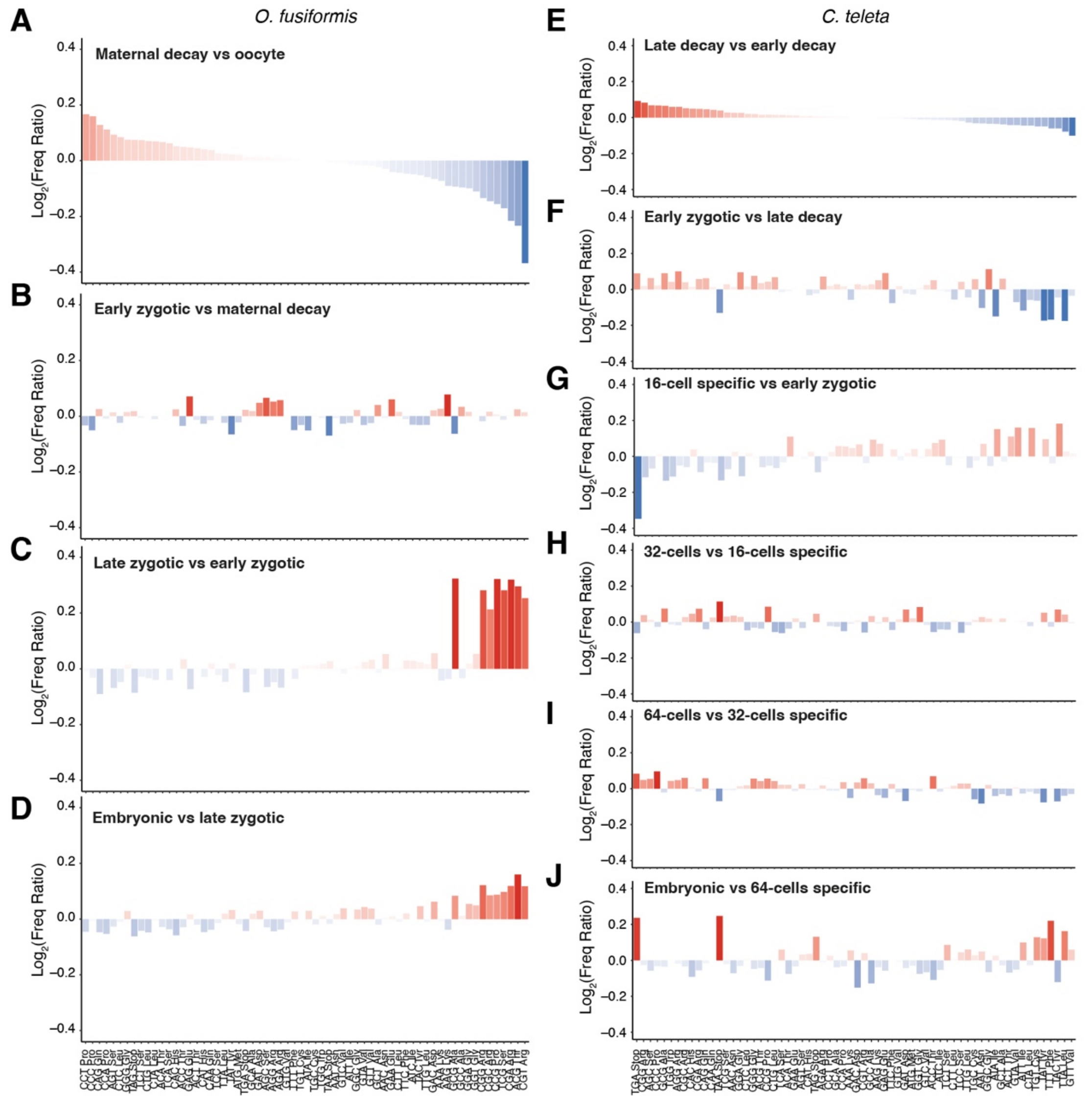

Appendix Figure S7 – Codon usage during spiral cleavage. (**A**–**J**) Bar plots indicating positive (red) or negative (blue) biases in codon usage between pairwise comparisons of clusters of temporally coregulated genes in *O. fusiformis* (**A**–**D**) and *C. teleta* (**E**–**J**). In *O. fusiformis*, codon usage distribution in oocyte-specific genes (**A**) differs from that of zygotic genes (**C**, **D**). These marked differences are not as evident in *C. teleta* (**E**–**J**).

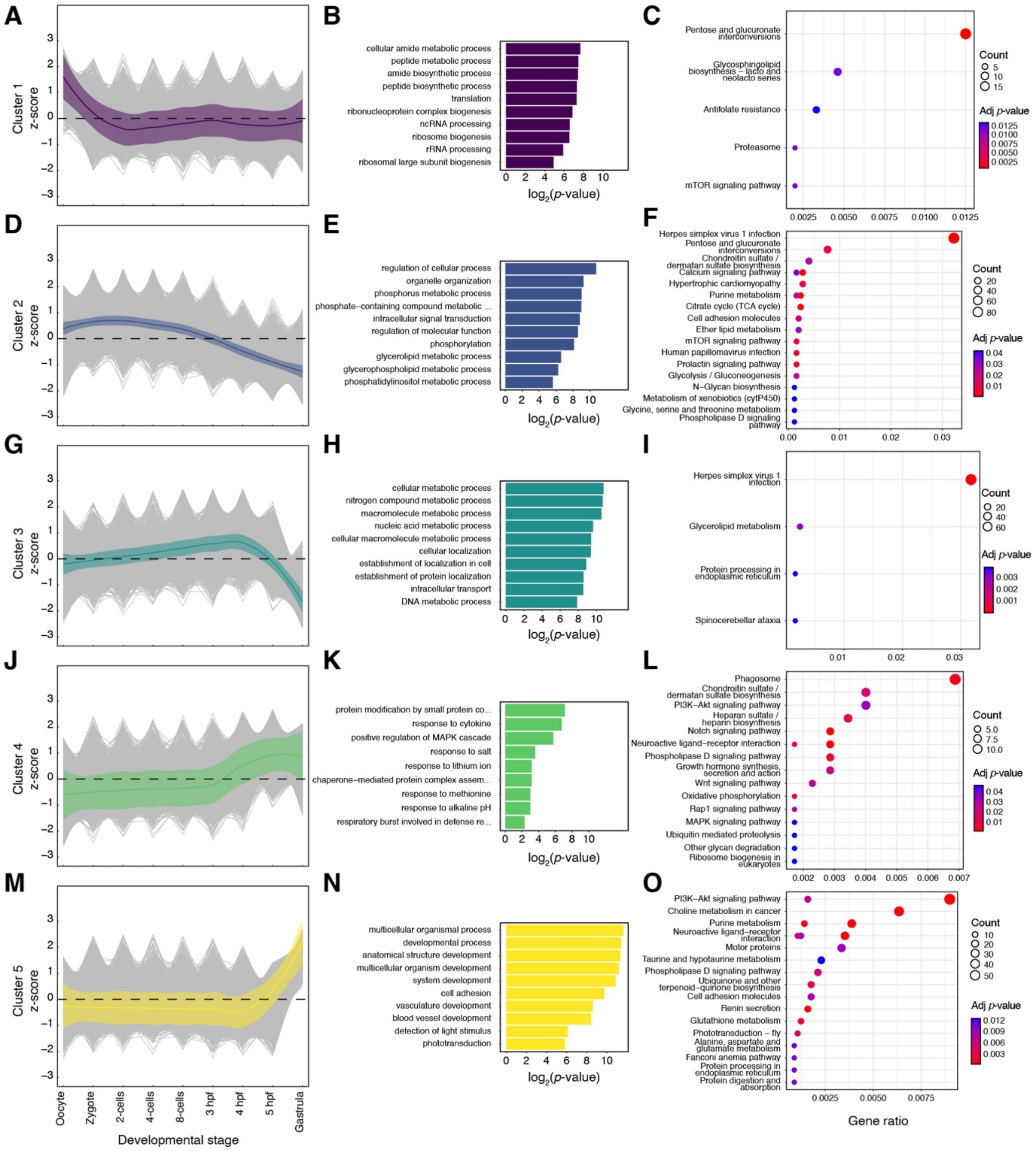

Appendix Figure S8 – Functional annotation of clusters of temporally co-regulated genes in *O. fusiformis*. (**A**, **D**, **G**, **J**, **M**) Gene-wise expression dynamics (grey lines) and locally estimated scatterplot smoothing (coloured lines) for each cluster of temporally coregulated genes during *O. fusiformis* spiral cleavage. Coloured shaded areas represent the standard error of the mean. (**B**, **E**, **H**, **K**, **N**) Bar plots indicating the top ten Gene Ontology (GO) categories amongst genes in each cluster. (**C**, **F**, **I**, **L**, **O**) KEGG enrichment plots for each cluster of coregulated genes during spiral cleavage in *O. fusiformis*.

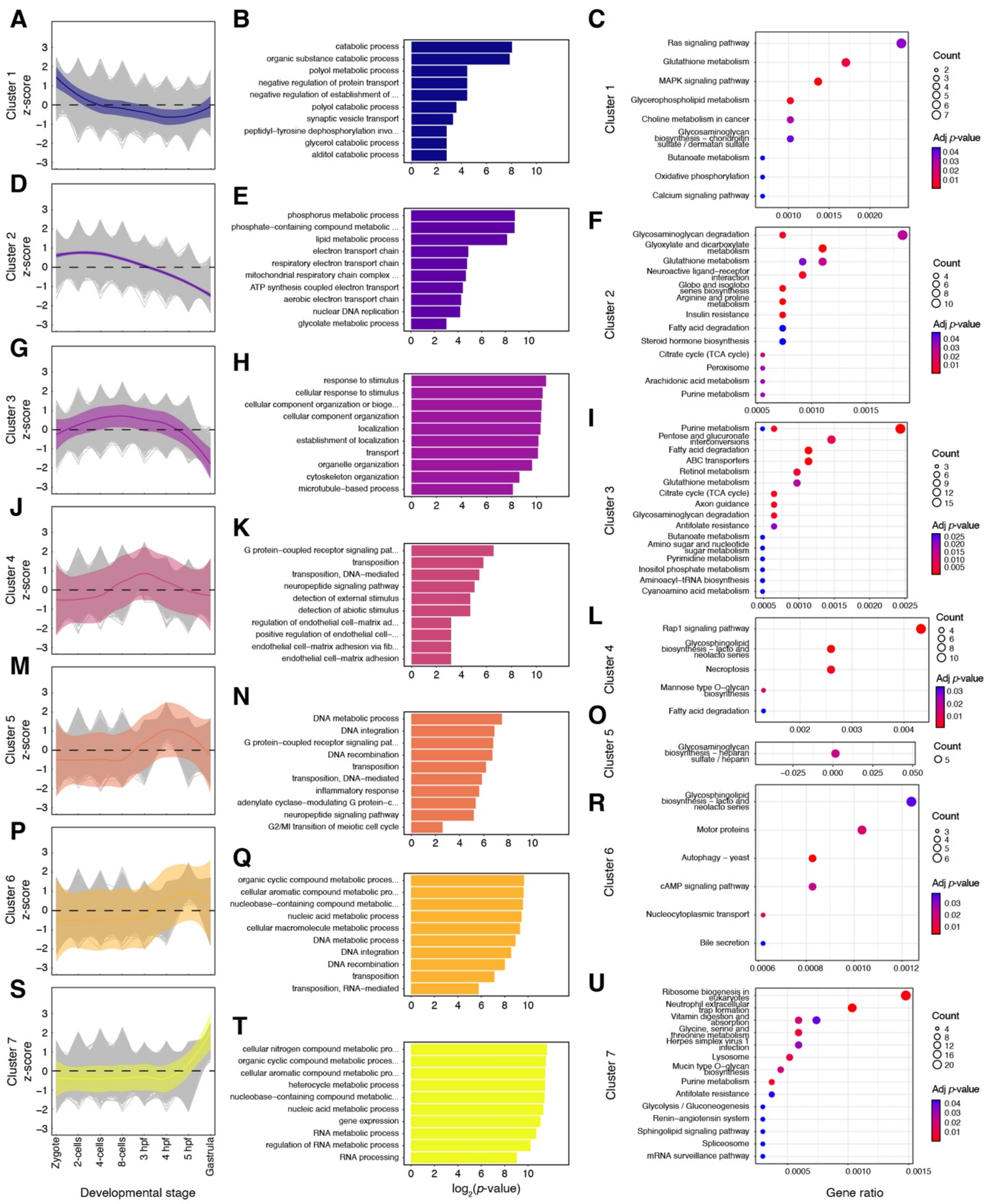

Appendix Figure S9 – Functional annotation of clusters of temporally co-regulated genes in *C. teleta*. (**A**, **D**, **G**, **J**, **M**, **P**, **S**) Gene-wise expression dynamics (grey lines) and locally estimated scatterplot smoothing (coloured lines) for each cluster of temporally coregulated genes during *C. teleta* spiral cleavage. Coloured shaded areas represent the standard error of the mean. (**B**, **E**, **H**, **K**, **N**, **Q**, **T**) Bar plots indicating the top ten Gene Ontology (GO)

categories amongst genes in each cluster. (**C**, **F**, **I**, **L**, **O**, **R**, **U**) KEGG enrichment plots for each cluster of coregulated genes during spiral cleavage in *C. teleta*.

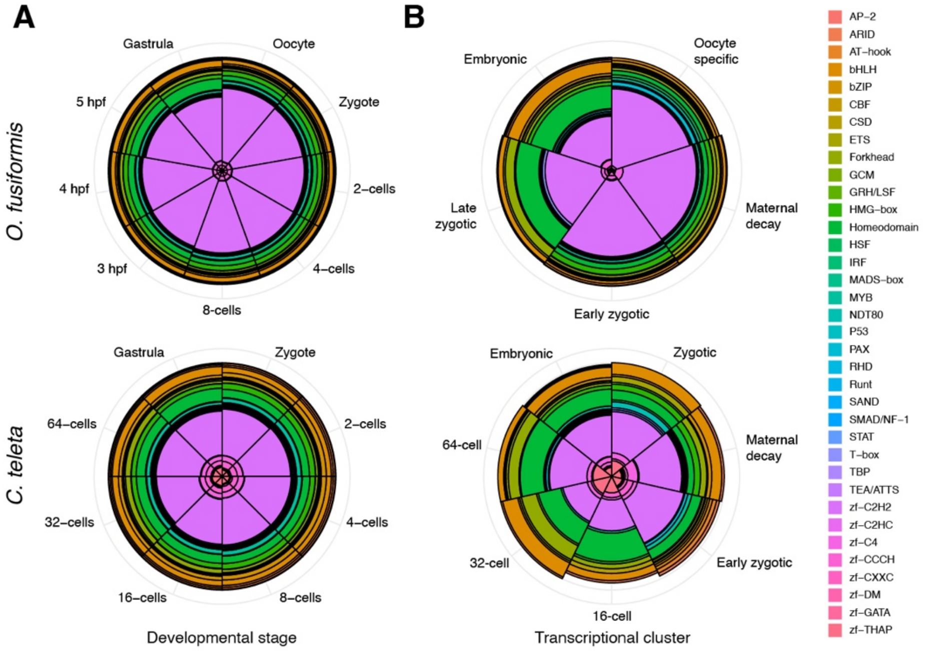

Appendix Figure S10 – The proportion of transcription factors during spiral cleavage. (**A**, **B**) Rose plots of transcription factor (TF) distribution (as a percentage) according to developmental time point (**A**) and cluster of coregulated genes (**B**).

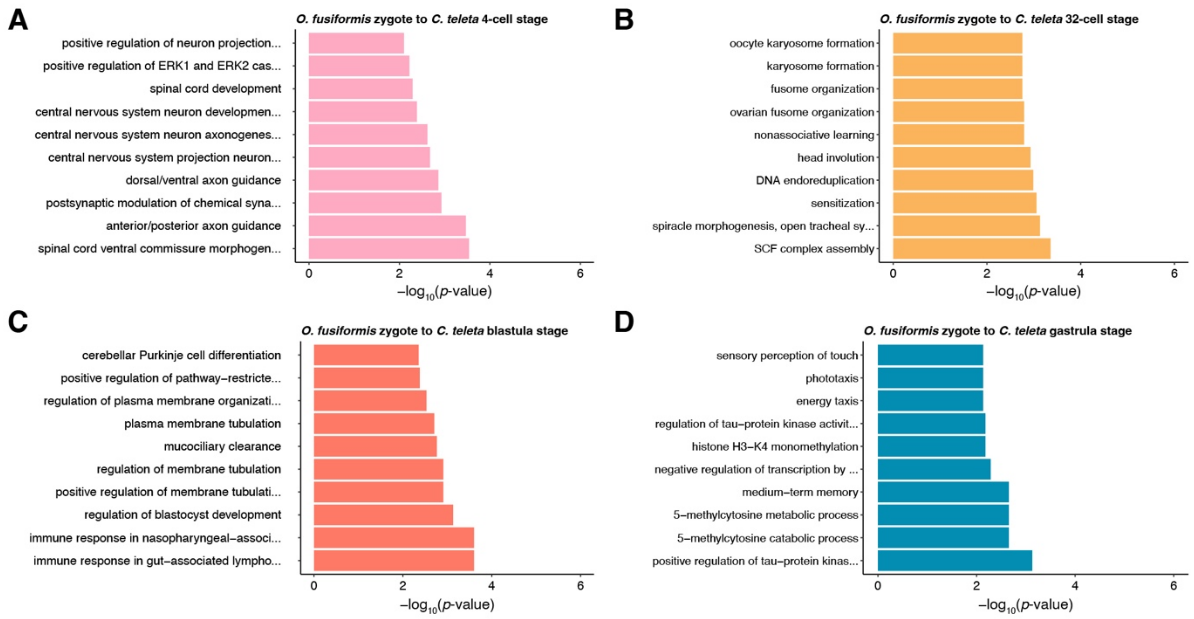

Appendix Figure S11 – Gene Ontology enrichment in maternal genes in *O. fusiformis*. (**A**–**D**) The bar plots indicate the top ten Gene Ontology (GO) categories amongst maternal genes in *O. fusiformis* that are expressed later in *C. teleta*.

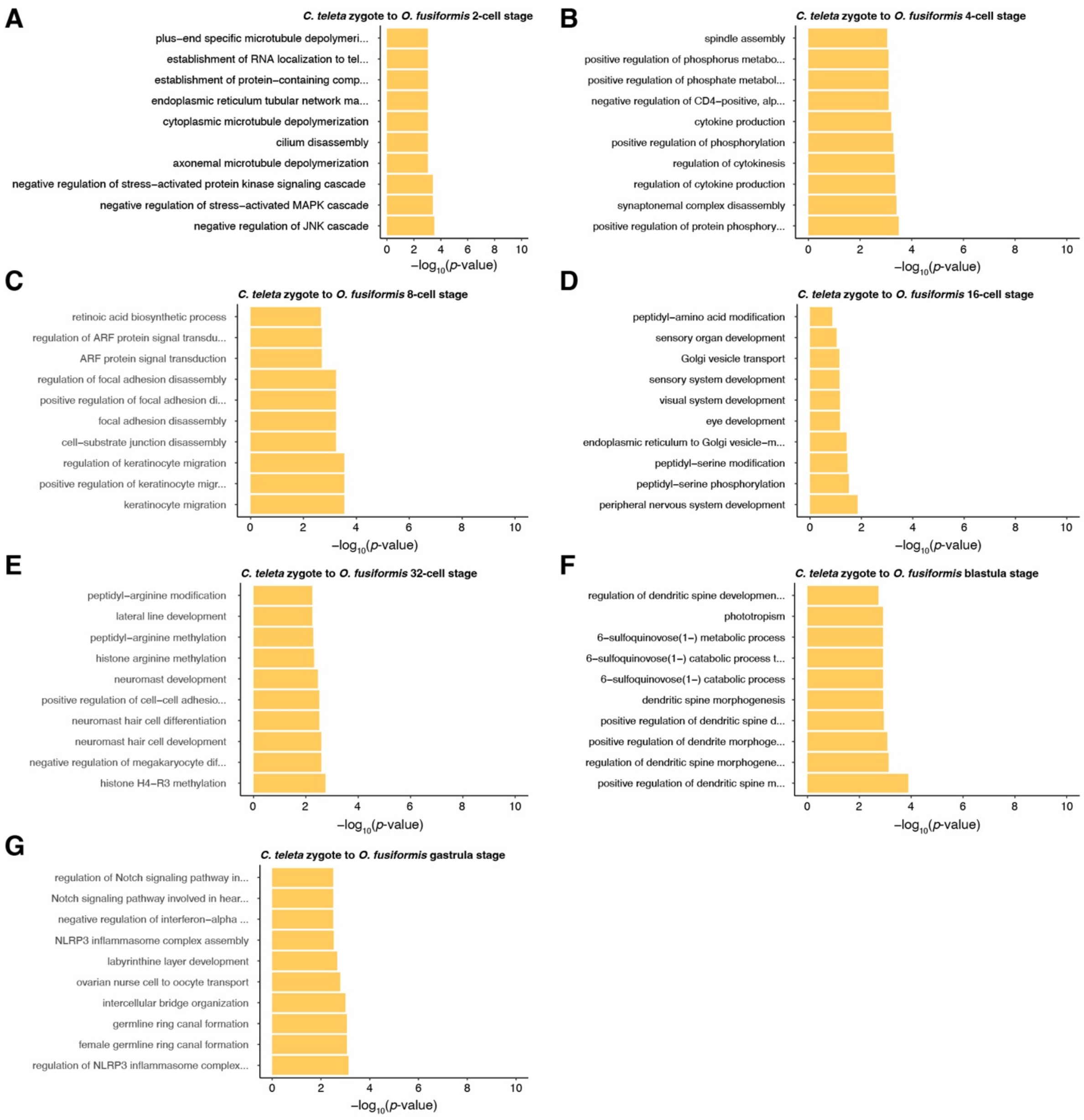

Appendix Figure S12 – Gene Ontology enrichment in maternal genes in *C. teleta*. (A–G) The bar plots indicate the top ten Gene Ontology (GO) categories amongst maternal genes in *C. teleta* that are expressed later in *O. fusiformis*.

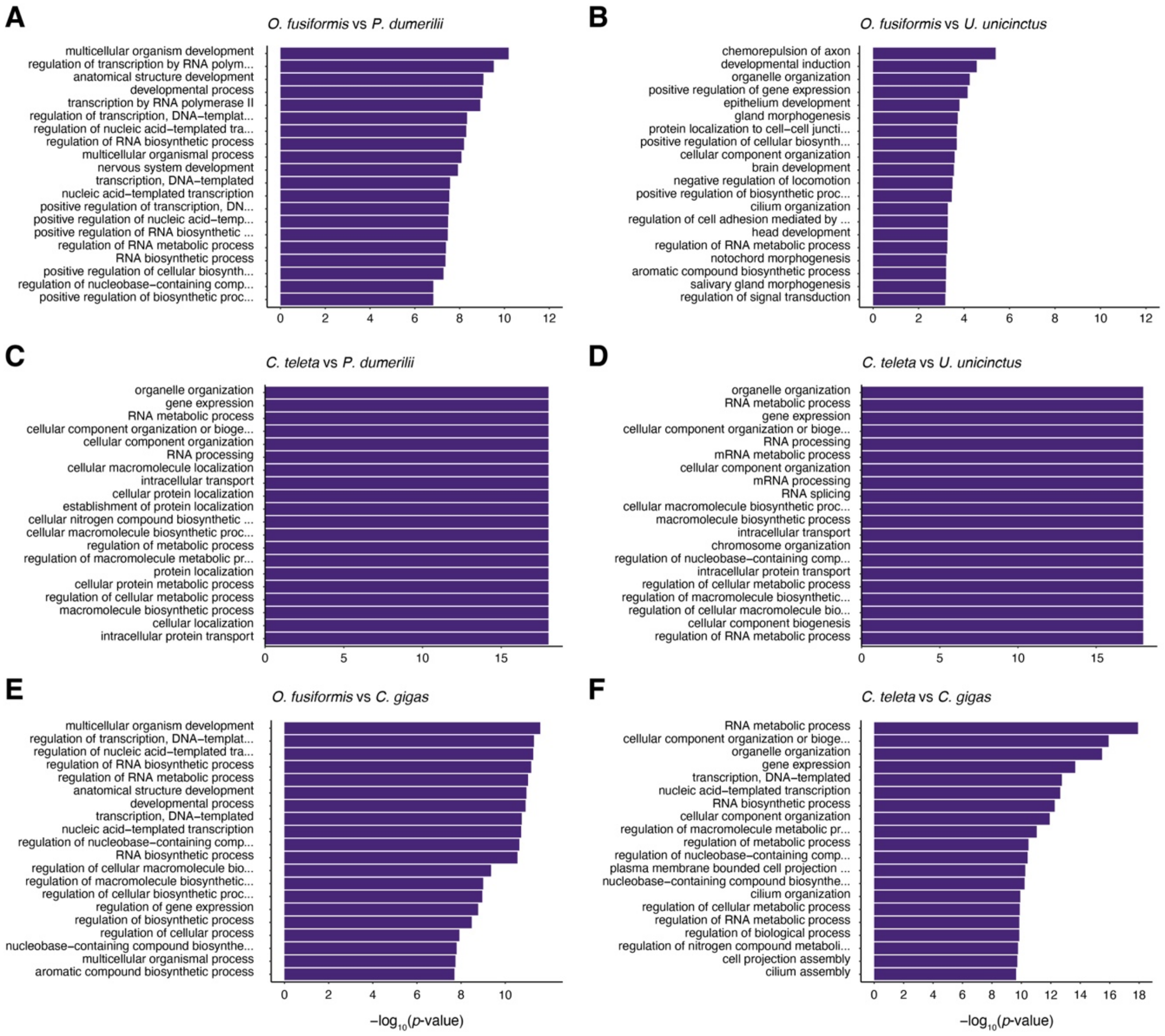

Appendix Figure S13 – Gene Ontology enrichment of genes exhibiting heterochronic shifts between different spiralian taxa. (**A**–**F**) The bar plots indicate the top twenty Gene Ontology (GO) categories amongst genes exhibiting heterochronic shift between *O. fusiformis* and *P. dumerilii* (**A**), *O. fusiformis* and *U. unicinctus* (**B**), *C. teleta* and *P. dumerilii* (**C**), *C. teleta* and *U. unicinctus* (**D**), *O. fusiformis* and *C. gigas* (**E**), and *C. teleta* and *C. gigas* (**F**).

Appendix Table S1 – Number of up- and downregulated genes during early embryogenesis in *O. fusiformis* and *C. teleta*.

Species	Comparison	Change	No. DEGs
O. fusiformis	Zygote vs Oocyte	Up	203
O. fusiformis	Zygote vs Oocyte	Down	290
O. fusiformis	2-cell vs Zygote	Up	9
O. fusiformis	2-cell vs Zygote	Down	12
O. fusiformis	4-cell vs 2-cell	Up	9
O. fusiformis	4-cell vs 2-cell	Down	7
O. fusiformis	8-cell vs 4-cell	Up	10
O. fusiformis	8-cell vs 4-cell	Down	12
O. fusiformis	3 hpf vs 8-cell	Up	78
O. fusiformis	3 hpf vs 8-cell	Down	59
O. fusiformis	4 hpf vs 3 hpf	Up	327
O. fusiformis	4 hpf vs 3 hpf	Down	70
O. fusiformis	5 hpf vs 4 hpf	Up	927
O. fusiformis	5 hpf vs 4 hpf	Down	263
O. fusiformis	Gastrula vs 5 hpf	Up	6853
O. fusiformis	Gastrula vs 5 hpf	Down	4359
C. teleta	2-cell vs Zygote	Up	527
C. teleta	2-cell vs Zygote	Down	1101
C. teleta	4-cell vs 2-cell	Up	36
C. teleta	4-cell vs 2-cell	Down	85
C. teleta	8-cell vs 4-cell	Up	125
C. teleta	8-cell vs 4-cell	Down	101
C. teleta	16-cell vs 8-cell	Up	1890
C. teleta	16-cell vs 8-cell	Down	200
C. teleta	32-cell vs 16-cell	Up	910
C. teleta	32-cell vs 16-cell	Down	189
C. teleta	64-cell vs 32-cell	Up	1767
C. teleta	64-cell vs 32-cell	Down	1078
C. teleta	Gastrula vs 64-cell	Up	8943
C. teleta	Gastrula vs 64-cell	Down	9302

Appendix Table S2 – Sequencing and mapping statistics for *O. fusiformis*.

sample	n_processed	n_pseudoaligned	n_unique	p_pseudoaligned	p_unique
active_oocyte_R1	42760709	25193265	20930034	58.9	48.9
active_oocyte_R2	41755797	23264926	19817215	55.7	47.5
1_cell__R1	41650434	24860001	20515843	59.7	49.3
1_cell__R2	47164566	29764456	24560476	63.1	52.1
2_cell__R1	44590774	26649171	21985675	59.8	49.3
2_cell__R2	43188381	26052299	21590273	60.3	50
4_cell__R1	45902725	27795905	22962786	60.6	50
4_cell__R2	49945828	30100090	24997113	60.3	50
8_cell__R1	45680904	27656772	22838790	60.5	50
8_cell__R2	49401610	30669669	25471137	62.1	51.6
3h_R1	47976852	27483311	22996563	57.3	47.9
3h_R2	44035927	25470773	21339058	57.8	48.5
4h_R1	45997615	27488530	23053719	59.8	50.1
4h_R2	52692310	33066709	27682661	62.8	52.5
blastula_R1	54631086	32769038	27544487	60	50.4
blastula_R2	46947697	30073063	25278898	64.1	53.8
gastrula_R1	52833442	28418664	24101891	53.8	45.6
gastrula_R2	49640037	30667038	25484641	61.8	51.3

Appendix Table S3 – Sequencing and mapping statistics for *C. teleta*.

sample	n_processed	n_pseudoaligned	n_unique	p_pseudoaligned	p_unique
Oocyte_R1	80469629	68149527	58738062	84.7	73
Oocyte_R2	78351191	67577503	58849376	86.2	75.1
1_cell__R1	77019707	65039014	57335827	84.4	74.4
1_cell__R2	74364267	65007348	57650126	87.4	77.5
2_cell__R1	71426889	61615313	54522772	86.3	76.3
2_cell__R2	86916744	75018864	66040149	86.3	76
4_cell__R1	62109990	53978101	47798965	86.9	77
4_cell__R2	76816071	66895058	59190944	87.1	77.1
8_cell__R1	73725921	64114773	56782998	87	77
8_cell__R2	74316006	64366750	56721096	86.6	76.3
16_cell__R1	77919164	67124863	59288563	86.1	76.1
16_cell__R2	76968528	66629889	58741125	86.6	76.3
32_cell__R1	76367828	65185678	57354350	85.4	75.1
32_cell__R2	73179550	62854031	55415446	85.9	75.7
64_cell__R1	76418625	65071080	56908303	85.2	74.5
64_cell__R2	72974608	62705285	54977463	85.9	75.3
gastrula_R1	78944044	66228561	57256746	83.9	72.5
gastrula_R2	76630568	65655886	57147861	85.7	74.6

Appendix Table S4 – Primers used to clone the validated transcription factors in *O. fusiformis* and *C. teleta*.

Primer	Sequence
Ofus_fjx1_F1(268)	ACCGAGTGAACTGCTGTAGA
Ofus_fjx1_F2(773)	ACCCGCTAGACAACACATAAA
Ofus_HNF4A_F(364)	GACACTGACATCTGTTGGGC
Ofus_HNF4A_R(1444)	GTGAAGTGCTGCTGATGAGG
Ofus_UNC-4_F(582)	GATCTCATTGAAAGCCGCGT
Ofus_UNC-4_R(1522)	CTTGCGTCTCGTTATGTGCA
Ofus_AP2_F(779)	GAGATGGGCACAGAAGGACT
Ofus_AP2_R(1886)	GCTGTTCAACACCGTTGCTA
Ofus_pax2/5/8_F(425)	AAAGAATCGTCGAGCTTGCC
Ofus_pax2/5/8_R(1498)	CGAGAGCAGGTTTGAGTTCG
Ofus_VSX2_F(692)	CCATTCATTCCACGAACGCT
Ofus_VSX2_R(1594)	CCATAGCAAAACACGTCCCC
Ofus_TBX2/3_F(786)	CAAACACGGCTTTCAGACCA
Ofus_TBX2/3_R(1860)	CGGTAAACTGGGACTTGCAC
Ofus_prop1_F(403)	TGGAATCAGCGTTCGCAAAA
Ofus_prop1_R(1249)	AGAGTGCAAATAATCCAACGGT
Ctel_AP2_F(292)	ACGTCGCTAGTTGAAGGTGA
Ctel_AP2_R(1158)	GCGAATCATGCACACAGACA
Ctel_FJX1_F(397)	CGCATCAACAACGACCAGAT
Ctel_FJX1_R(1247)	CCGTTGATGGTTAGGGGAGA
Ctel_VSX2_F(559)	AGACCGAGGCAAGGATGAAA
Ctel_VSX2_R(1713)	TATCGTGACGGCTCAGTGAA
Ctel_UNC-4_F(598)	AGATGGCCTACACGATGGAG
Ctel_UNC-4_R(1535)	CATTGGACTTGTGGTTGGGG
Ctel_HNF4G_F(338)	TGTGTACTCGTGTCGGTTCA
Ctel_HNF4G_R(1178)	GCCAAATAGCTTCGCCACTT
Ctel_TBX2/3_F(576)	CTCGATGCACAAGTACCAGC
Ctel_TBX2/3_R(1484)	CCAGGAGACAGCTGAGAGAG
Ctel_pax2/5/8_F(514)	CTAACGGAGAGCGTGTGTTC
Ctel_pax2/5/8_R(1538)	GTGGATGTGAACTGTTGCGT
Ctel_prop1_F(605)	CAAGTACCGGAAGCAGGAGA
Ctel_prop1_R(1437)	CACCCAGGAGCTAGGAATGT
