# Supplementary figures and images for: Cell fate specification modes shape transcriptome evolution in the highly conserved spiral cleavage
Source: EMBO Rep. 2025 Sep 4;26(20):5088–114. doi: 10.1038/s44319-025-00569-4 (PMC12550047; doi:10.1038/s44319-025-00569-4)

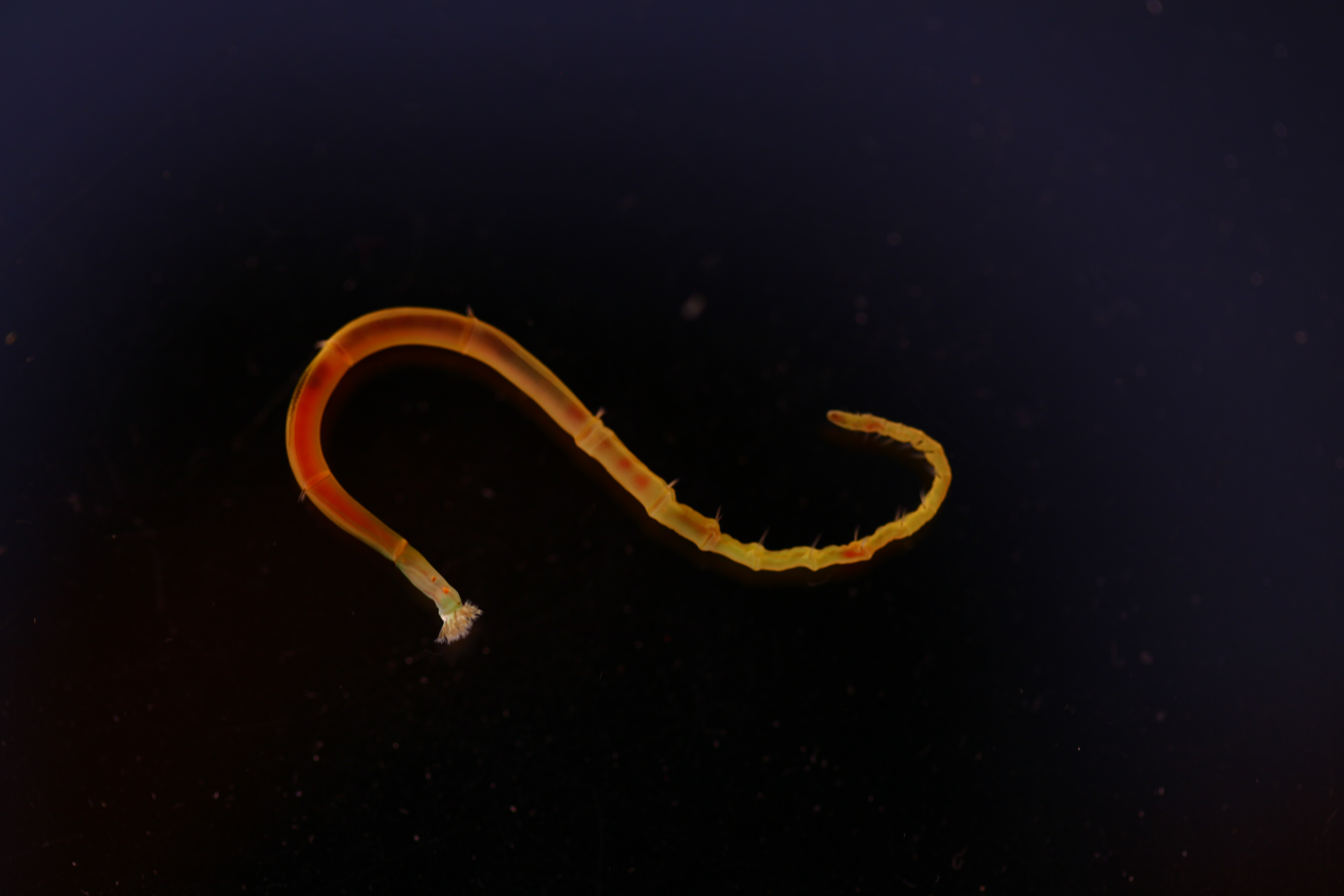

Supplement: Supplementary file 47 — Source data Fig. 1 [file 44319_2025_569_MOESM47_ESM.zip › Figure1B/IMG_5702.JPG]

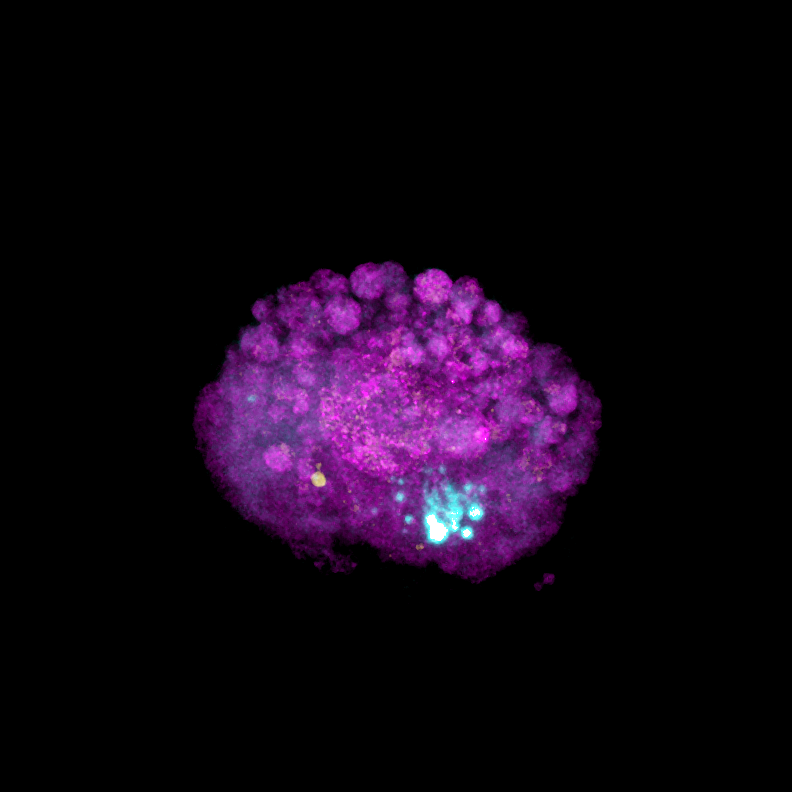

Supplement: Supplementary file 48 — Source data Fig. 3 [file 44319_2025_569_MOESM48_ESM.zip › Figure3A/2to5hOwenia.tif]

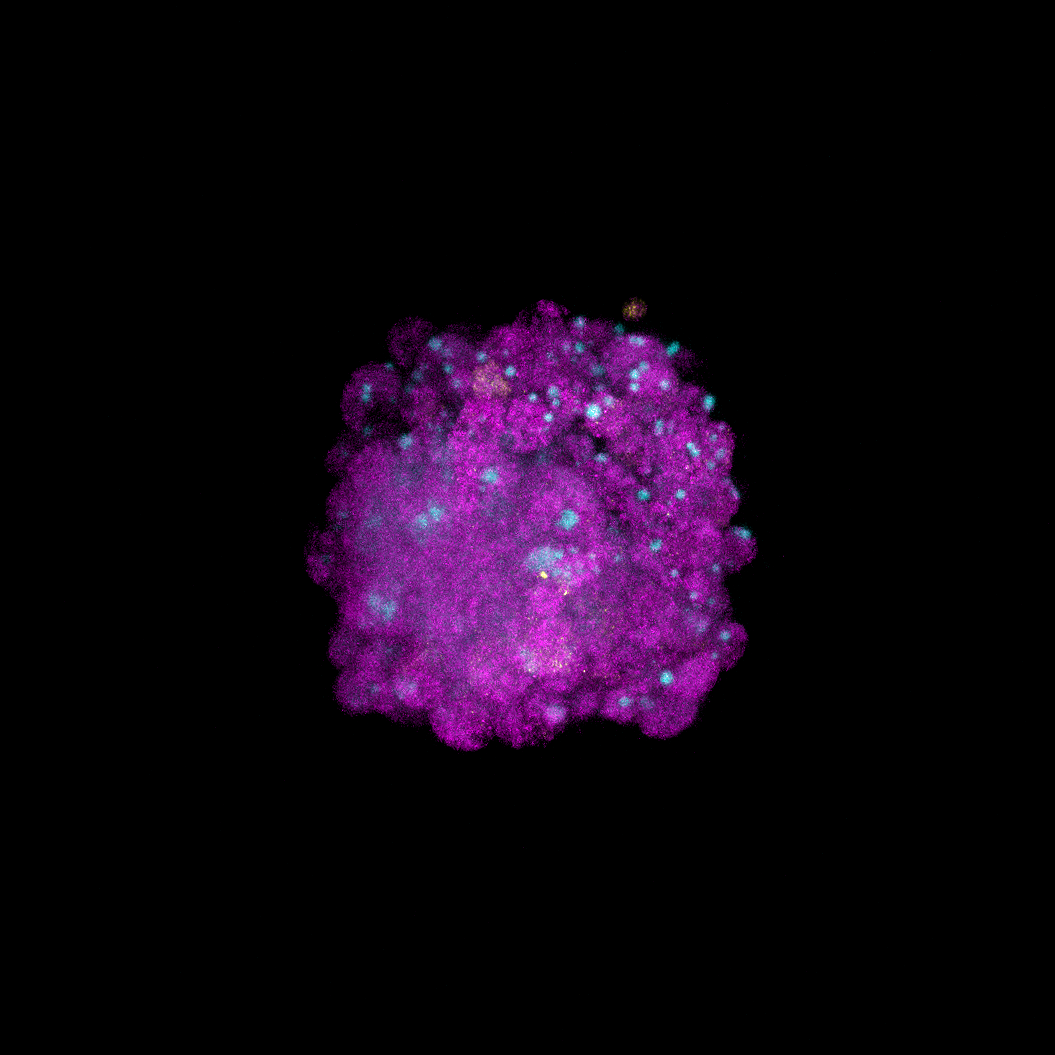

Supplement: Supplementary file 48 — Source data Fig. 3 [file 44319_2025_569_MOESM48_ESM.zip › Figure3A/2to6hOwenia.tif]
