# Supplementary material for: Cell fate specification modes shape transcriptome evolution in the highly conserved spiral cleavage
Source: EMBO Rep. 2025 Sep 4;26(20):5088–114. doi: 10.1038/s44319-025-00569-4 (PMC12550047; doi:10.1038/s44319-025-00569-4)
Supplement: Supplementary file 49 — Expanded View Figures [file 44319_2025_569_MOESM49_ESM.pdf]

Expanded View Figures

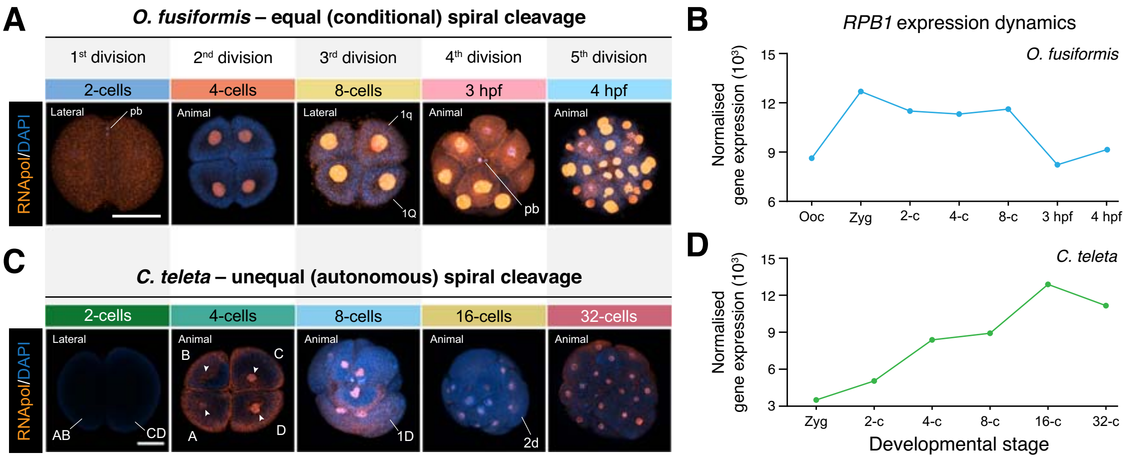

Figure EV1. Dynamics of RNA polymerase II nuclearisation during spiral cleavage.

(**A**, **C**) Z-projections of confocal stacks of embryos of *O. fusiformis* (**A**) and *C. teleta* (**C**) from the 2-cell stage to 4 h post-fertilisation (hpf) or the 32-cell stage. RNA polymerase II localises to the nuclei from the 4-cell stage onwards in both annelids. In *C. teleta*, the nuclearisation is more intense in the C and D blastomeres than in the A and B cells. (**B**, **D**) Expression dynamics of the *RPB1* gene (largest subunit of the RNA polymerase II, recognised by the antibody used in **A** and **C**) in *O. fusiformis* (**B**) and *C. teleta* (**D**). In the two annelids, RPB1 is a highly abundant maternal gene. Gene expression values are the average of two biological replicates.

 © The Author(s)

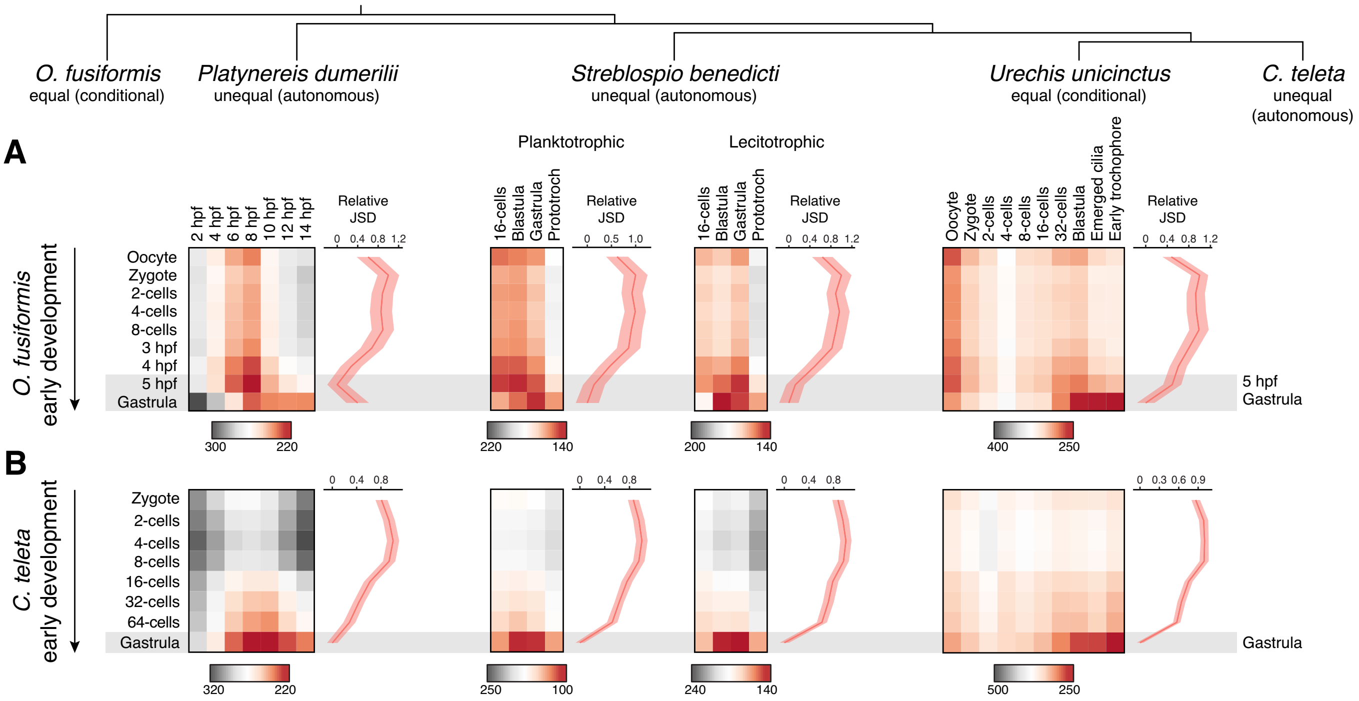

Figure EV2. Transcriptomic dynamics during spiral cleavage in *Annelida*.

(**A**, **B**) Jensen-Shannon transcriptomic divergence during the spiral cleavage between *O. fusiformis* (**A**) and *C. teleta* (**B**) and four other annelid species with publicly available transcriptomic resources covering at least one cleavage stage and the gastrula stage. In all cases, the point of maximal transcriptomic similarity occurs at the late cleavage and gastrulation (grey horizontal bar).

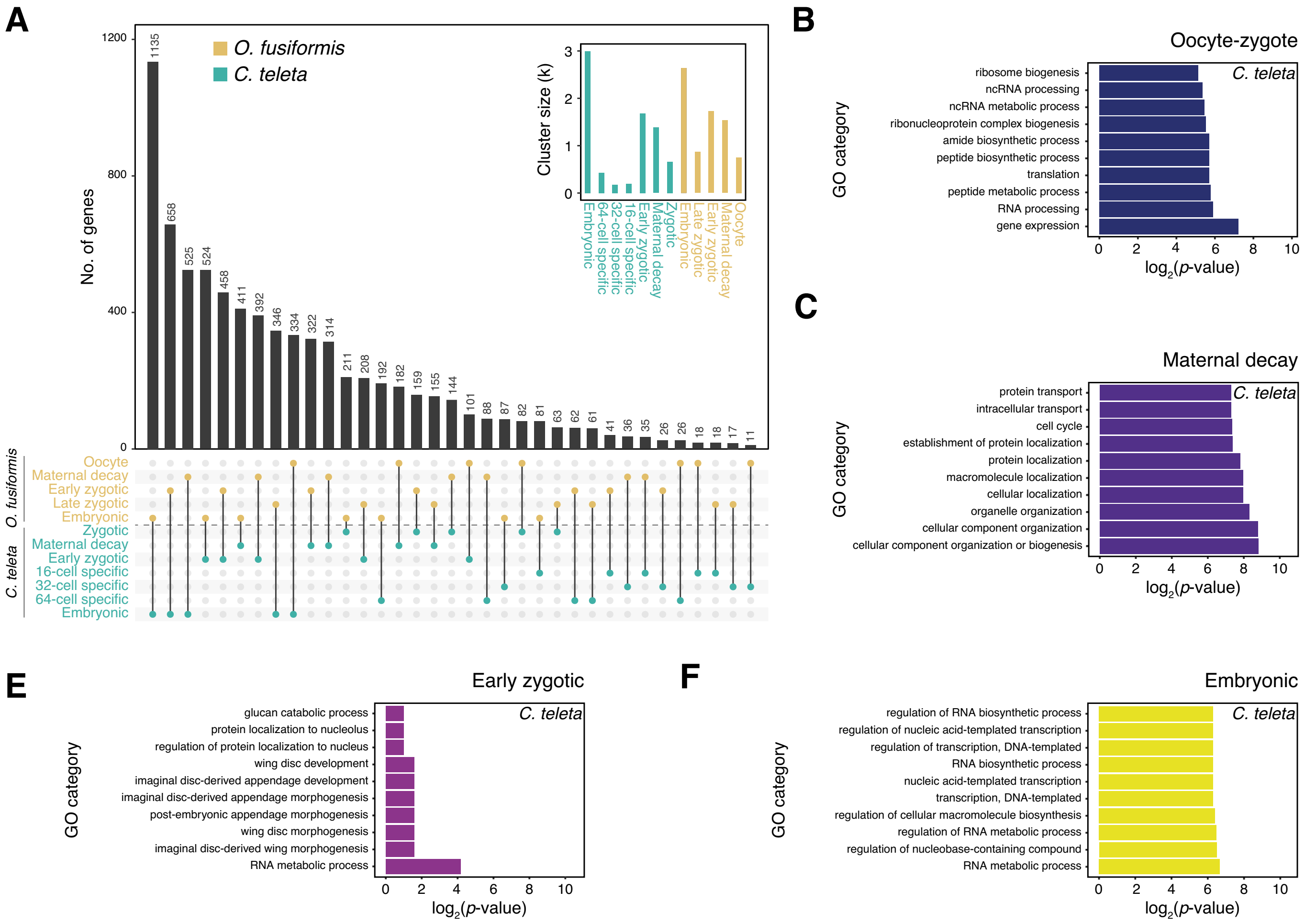

Figure EV3. The comparison of clusters of temporally coregulated genes between conditional and autonomous spiral cleavage.

(**A**) Upset plot indicating the number of shared one-to-one orthologs between clusters of temporally coregulated genes. The inset indicates the total number of genes in each cluster. (**B–F**) Bar plots indicating the top ten Gene Ontology (GO) categories amongst shared orthologous genes in the oocyte/zygote, maternal decay, early zygotic, and embryonic clusters, according to the GO annotation for the *C. teleta* ortholog. *P* values were computed from upper-tail Fisher's exact tests.

 © The Author(s)

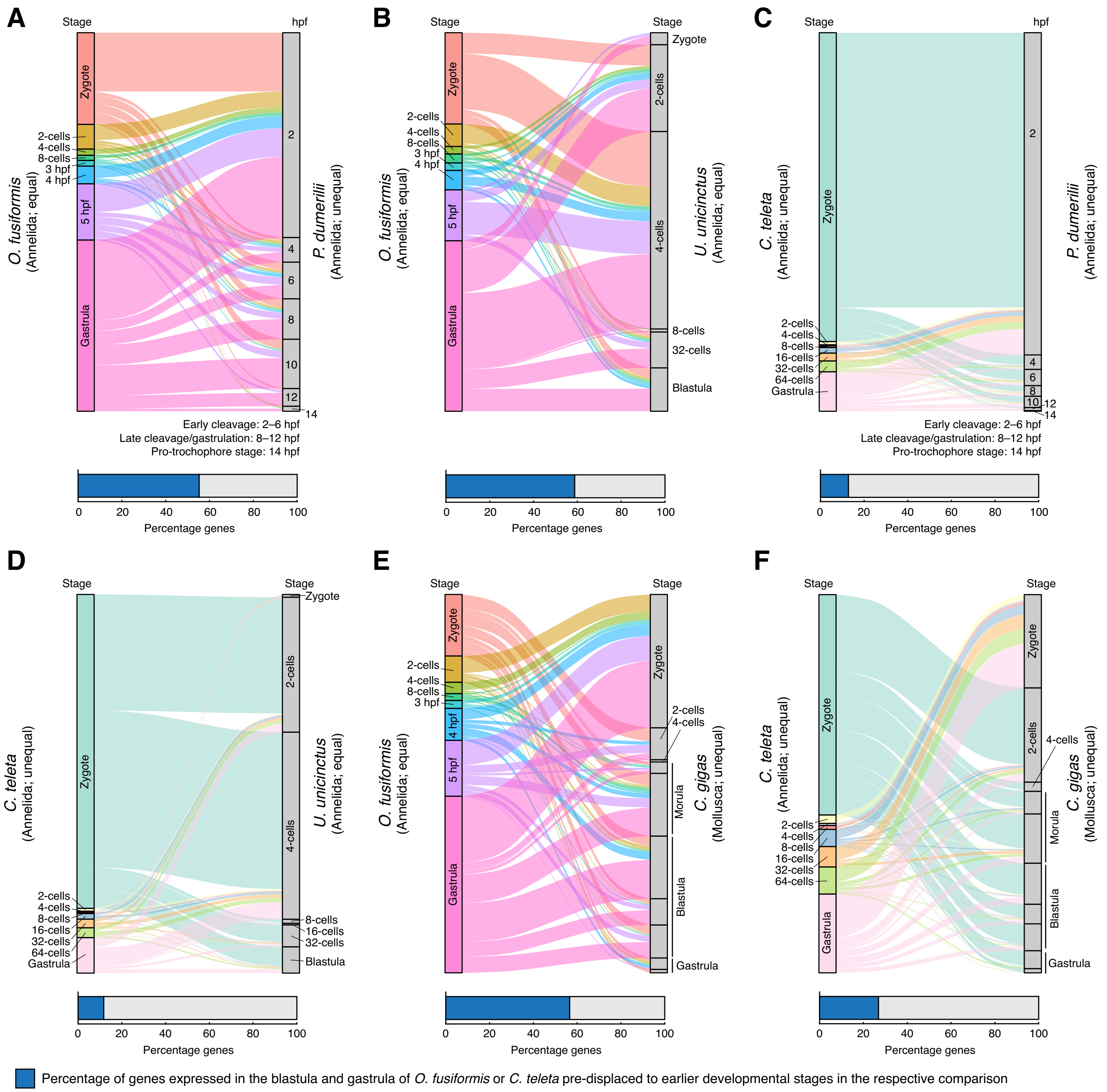

Percentage of genes expressed in the blastula and gastrula of *O. fusiformis* or *C. teleta* pre-displaced to earlier developmental stages in the respective comparison

Figure EV4. Heterochronic shifts in gene expression between selected molluscan and annelid species.

(**A–F**) Alluvial plots depicting the comparative deployment of one-to-one orthologs exhibiting shifts in temporal activation during spiral cleavage between *O. fusiformis* and *P. dumerilii* (**A**), *O. fusiformis* and *U. unicinctus* (**B**), *C. teleta* and *P. dumerilii* (**C**), *C. teleta* and *U. unicinctus* (**D**), *O. fusiformis* and *C. gigas* (**E**), and *C. teleta* and *C. gigas* (**F**). Generally, more genes shift from late cleavage stages in *O. fusiformis* to early stages in other species than when *C. teleta* is included in equivalent comparisons.

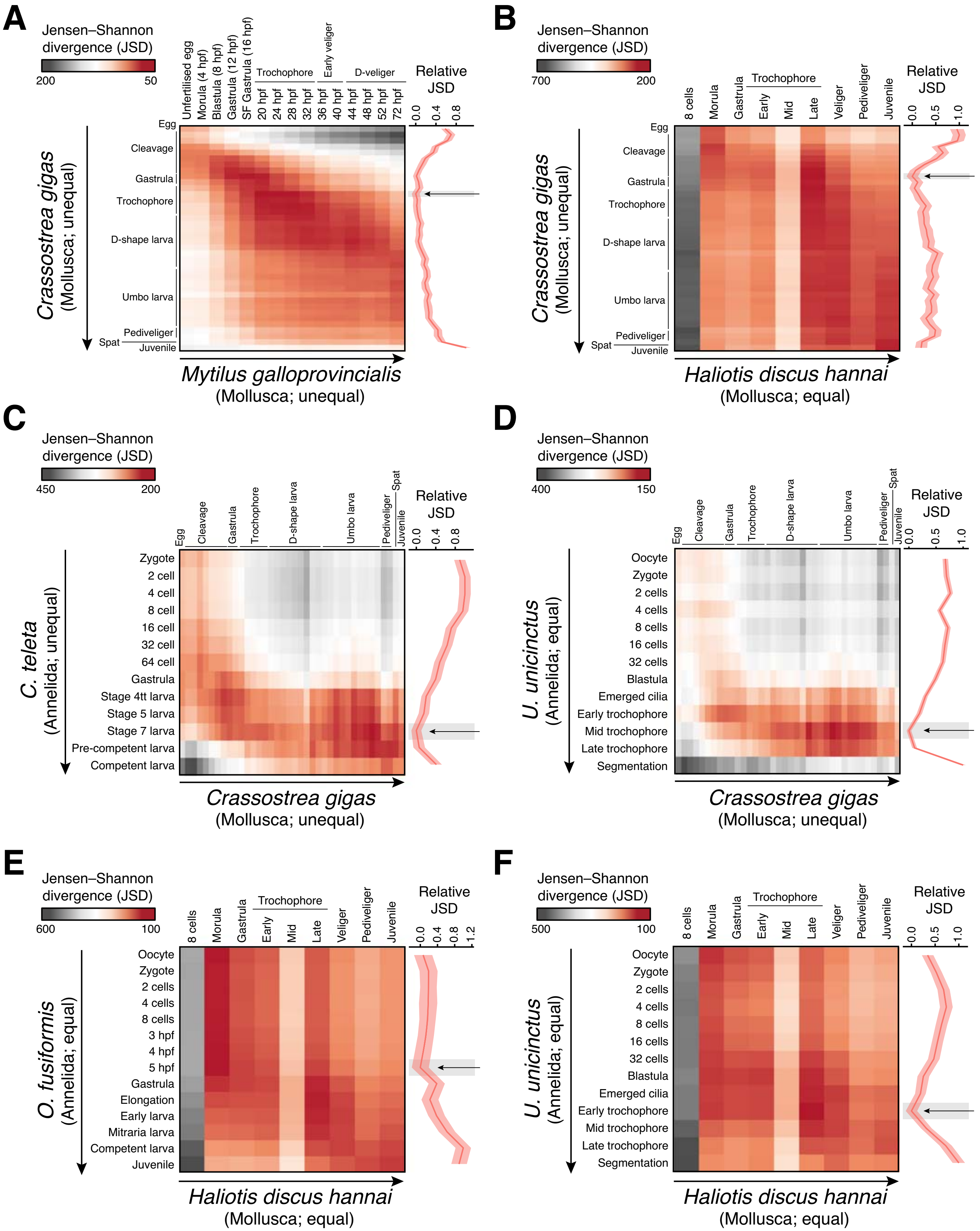
A
Jensen–Shannon divergence (JSD)
200
50
Unfertilised egg
Morula (4 hpf)
Blastula (8 hpf)
Gastrula (12 hpf)
SF Gastrula (16 hpf)
Trochophore
20 hpf
24 hpf
28 hpf
32 hpf
36 hpf
Early veliger
D-veliger
40 hpf
44 hpf
48 hpf
52 hpf
72 hpf
Relative JSD
0.0
0.4
0.8
Egg
Cleavage
Gastrula
Trochophore
D-shape larva
Umbo larva
Pediveliger
Spat
Juvenile
Crassostrea gigas
(Mollusca; unequal)
Mytilus galloprovincialis
(Mollusca; unequal)
B
Jensen–Shannon divergence (JSD)
700
200
8 cells
Morula
Gastrula
Trochophore
Early
Mid
Late
Veliger
Pediveliger
Juvenile
Relative JSD
0.0
0.5
1.0
Egg
Cleavage
Gastrula
Trochophore
D-shape larva
Umbo larva
Pediveliger
Spat
Juvenile
Crassostrea gigas
(Mollusca; unequal)
Haliotis discus hannai
(Mollusca; equal)
C
Jensen–Shannon divergence (JSD)
450
200
Egg
Cleavage
Gastrula
Trochophore
D-shape larva
Umbo larva
Pediveliger
Spat
Juvenile
Relative JSD
0.0
0.4
0.8
Zygote
2 cell
4 cell
8 cell
16 cell
32 cell
64 cell
Gastrula
Stage 4tt larva
Stage 5 larva
Stage 7 larva
Pre-competent larva
Competent larva
C. teleta
(Annelida; unequal)
Crassostrea gigas
(Mollusca; unequal)
D
Jensen–Shannon divergence (JSD)
400
150
Egg
Cleavage
Gastrula
Trochophore
D-shape larva
Umbo larva
Pediveliger
Spat
Juvenile
Relative JSD
0.0
0.5
1.0
Oocyte
Zygote
2 cells
4 cells
8 cells
16 cells
32 cells
Blastula
Emerged cilia
Early trochophore
Mid trochophore
Late trochophore
Segmentation
U. unicinctus
(Annelida; equal)
Crassostrea gigas
(Mollusca; unequal)
E
Jensen–Shannon divergence (JSD)
600
100
8 cells
Morula
Gastrula
Trochophore
Early
Mid
Late
Veliger
Pediveliger
Juvenile
Relative JSD
0.0
0.4
0.8
1.2
Oocyte
Zygote
2 cells
4 cells
8 cells
3 hpf
4 hpf
5 hpf
Gastrula
Elongation
Early larva
Mitraria larva
Competent larva
Juvenile
O. fusiformis
(Annelida; equal)
Haliotis discus hannai
(Mollusca; equal)
F
Jensen–Shannon divergence (JSD)
500
100
8 cells
Morula
Gastrula
Trochophore
Early
Mid
Late
Veliger
Pediveliger
Juvenile
Relative JSD
0.0
0.5
1.0
Oocyte
Zygote
2 cells
4 cells
8 cells
16 cells
32 cells
Blastula
Emerged cilia
Early trochophore
Mid trochophore
Late trochophore
Segmentation
U. unicinctus
(Annelida; equal)
Haliotis discus hannai
(Mollusca; equal)

 © The Author(s)

Figure EV5. Transcriptional similarity between Molluscan and Annelid development.

(A–F) Jensen-Shannon transcriptomic divergence between all possible inter-species pairwise comparisons during the entire life cycle, from oocyte or cleavage to juvenile or competent larva, between molluscan and annelid species with a high-resolution time course. In intra-phylum comparisons, the stages of maximal similarity are at or around gastrulation. In contrast, in inter-phylum comparisons, the larval stages are more transcriptionally similar (except for the *O. fusiformis* versus *Haliotis discus hannai* comparisons, which might be due to the poor quality of the molluscan dataset).

© The Author(s)
